# The Value and Impacts of Academic Public Health Departments

**DOI:** 10.1146/annurev-publhealth-071421-031614

**Published:** 2022-10-20

**Authors:** Paul C. Erwin, Julie H. Grubaugh, Stephanie Mazzucca-Ragan, Ross C. Brownson

**Affiliations:** 1 School of Public Health, University of Alabama at Birmingham, Birmingham, Alabama, USA; 2 Department of Public Health, University of Tennessee, Knoxville, Tennessee, USA; 3 Brown School, Washington University in St. Louis, St. Louis, Missouri, USA; 4 Department of Surgery, Division of Public Health Sciences; and Alvin J. Siteman Cancer Center, Washington University School of Medicine; Washington University in St. Louis, St. Louis, Missouri, USA

**Keywords:** academic health department, AHD, public health, academic practice partnership, evidence-based decision-making, COVID-19, health equity

## Abstract

The academic health department (AHD) is a partnership between an academic institution and a governmental health agency. These partnerships are meant to provide mutual benefits that include opportunities for student field placements and internships, practice-informed curriculum, and practice-based research. The term academic health department dates back only to 2000, although there are several examples of academic–practice partnerships prior to that date. In addition to AHDs that have been established over the past two decades, other forms of academic–practice engagement provide similar mutual benefits, such as prevention research centers and public health training centers. Current research on AHDs explores how these partnerships matter regarding the outputs, outcomes, and impacts of the units that comprise them. This review also considers the most recent perspectives on how AHDs have responded to the COVID-19 pandemic and how they might advance public health’s efforts to address structural racism and promote health equity.

## INTRODUCTION

The academic public health department [or academic health department (AHD)] is defined as “an arrangement between an academic institution and a governmental public health agency that provides mutual benefits in teaching, research, and service, with academia informing the practice of public health, and the governmental public health agency informing the academic program” (21; 26, p. 270). The academic institution may be a health professions–focused school or program (e.g., a school of public health, medicine, or nursing), a department within a school, or even a community college that is building practice partnerships for future employment opportunities. The public health agency is typically a local (city or county) or state health department but may also be a tribal, territorial, or even federal public health agency. The relationship may range from informal and relatively passive to formal, active partnerships with a signed memorandum of understanding that describes the sharing of human, physical, or financial resources. The AHD partnership is meant to provide collaborative opportunities across academia and practice, involving field practice experiences for students (which may lead to employment), practice-based research, and public health practice workforce development, culminating in practice-informed teaching and academic-informed practice.

Although academic–public health practice partnerships date back several decades, the term academic health department first appears in a 2000 article by C. William Keck (39), who, as simultaneously the Director of the City of Akron Health Department and the Chair of the Department of Community Health Sciences at Northeastern Ohio Universities College of Medicine (now renamed the Department of Family and Community Medicine and the Northeast Ohio Medical University, respectively), was the very embodiment of the AHD partnership. Keck likened this arrangement to the public health corollary of the relationships between a teaching hospital and a school of medicine in academic medical centers. The landmark 1988 report from the Institute of Medicine (IOM, now the National Academy of Medicine), *The Future of Public Health* (38), gave new impetus for more closely aligning the academic training of public health professionals and the actual practice of public health in governmental agencies.

Since 2000, there has been a growing realization of the mutual value that academic–practice partnerships can have. Special themed issues of peer-reviewed journals, such as those of the *Journal of Public Health Management and Practice*, have provided the venue for scholarly attention to AHDs, while the establishment of the AHD Learning Community, a virtual community established in 2011 by the Council on Linkages Between Academia and Public Health Practice and staffed by the Public Health Foundation, has offered significant support to both practitioners and academicians in establishing new AHD partnerships (60). Our purpose in this review is to (*a*) describe the history and scope of academic–public health practice partnerships; (*b*) summarize both the qualitative and quantitative scholarly work on AHDs; (*c*) explore the function and value of AHDs in the context of the COVID-19 pandemic, as well as from a health equity lens; and (*d*) provide a road map for future research on AHDs. While our review focuses on literature and experiences from the United States, other countries have undertaken similar efforts (see the sidebar titled AHD Examples from Other Countries).

### The Roots of Modern-Day Academic–Practice Partnerships

Presaging the dual role that Keck played in the 1980s and 1990s, the academic–practice partnership between Columbia University and the New York City Department of Health began in the early twentieth century, when the dean of the Columbia School of Public Health and the commissioner of health were the same individual and the institutions shared space for both classrooms and clinics (20). In 1940, the School of Public Health and the city health department jointly developed the Washington Heights District Health Station, where public health students at Columbia took classes in the same place where the community obtained health care (34). Greene et al. (34) described the long-standing (and still current) Public Health/Preventive Medicine Residency Program run collaboratively by the health department and the School of Public Health, where many New York City Department of Health senior staff received their training (https://www1.nyc.gov/site/doh/about/employment/residency-program.page). For several years, beginning in 1997, the School of Public Health provided the Community Scholars Program in partnership with the New York state and city health departments, in which health department employees received up to 75% tuition support to obtain a Master of Public Health degree. Continuing this partnership, in the wake of the terrorist attacks on September 11, 2001, the National Center for Disaster Preparedness was established at Columbia’s Mailman School of Public Health, and the school subsequently partnered with the New York City Department of Health and Mental Hygiene to provide student exposure to clinical public heath, to establish a public health continuing education lecture series for health department employees, to facilitate the appointment of public health department professional staff to the school’s faculty, and to identify School of Public Health faculty to work at the health department to enhance the academic aspects of its clinical public health programs (20). This work, described as a “model academic health department” (20), currently includes the Health Research Training Program (HRTP), which provides research and practicum opportunities for health department staff and current public health students (https://www1.nyc.gov/site/doh/about/employment/hrtp-internship.page).

In 1932, the Johns Hopkins School of Hygiene and Public Health and the Baltimore City Health Department partnered to establish the Eastern Health District, which, as described by public health historian Elizabeth Fee, “provided data for researchers studying community health problems; learning opportunities for students in medicine, nursing, and public health administration; and a wide array of clinical and public health services to urban residents” (29, p. 735). The exchange between school faculty and students with health department employees was multifaceted, including Master of Public Health degree candidates spending a total of six mornings observing Health District activities as part of a required health administration course; senior medical students spending two mornings a week in Health District clinics; newly employed health department nurses receiving public health nursing training; and the Health District’s health officer and nursing supervisor teaching public health administration at the School of Public Health. The Health District focused attention on maternal and child health, mental health, sexually transmitted infections, and community-based research and teaching. The Health District’s monthly surveys on chronic illnesses beginning in 1938 were among the first studies to chart the incidence of noncommunicable diseases. By fielding multiple rounds of surveys of households within the catchment area over several years, the Health District compiled data on 60,000 people in 15,000 families, providing Hopkins’s researchers with a rich data set for study. Through this academic–practice partnership, new discoveries were made in the development of infant formula, the community-based approach to mental health, treatment for tuberculosis and syphilis, prenatal care, and well-child care. Although levels of collaboration waxed and waned after the first two decades of activities, the academic–practice engagement at the Eastern Health District entered the 1980s and 1990s by including services for, and research on, HIV/AIDS and remains as an active partnership site that provides services for sexual health, dental care, tuberculosis, and immunization clinics. Like the Washington Heights District Health Station in New York, the Eastern Health District in Baltimore provided an early powerful model for what an AHD partnership could become.

### The Modern-Day Impetus for Academic–Practice Linkages

As mentioned earlier, the IOM’s 1988 report *The Future of Public Health* was the most important impetus for changing the relationships between academia and practice. The report had described the “isolation” of academic public health from public health practice and provided several recommendations for ameliorating these relationships (38). In response to the report, Johns Hopkins University convened the Public Health Faculty/Agency Forum (also known as The Forum) as a means for translating the IOM’s recommendations on the engagement between academia and public health practice. Members of the Public Health Faculty/Agency Forum included the Association of Schools of Public Health (ASPH), the American Public Health Association (APHA), the Health Resources and Services Administration (HRSA), and the Centers for Disease Control and Prevention (CDC). An early product of this work was the establishment of the Center for the Development of Public Health Practice at the University of Illinois at Chicago (UIC) in 1991 (36). To document the extent of collaboration between academia and practice, even before the term academic health department was used, Arden Handler, Barney Turnock, and colleagues from UIC conducted multiple rounds of surveys of state health agencies and accredited schools of public health throughout the 1990s. The team surveyed all accredited schools of public health—27 at that time—and all 50 state health agencies, as well as public health agencies in the District of Columbia, Puerto Rico, and the US Virgin Islands. Baseline surveys were conducted in 1992, with follow-up surveys in 1993, 1994, and 1996, and a final report was published in 1999.

The baseline survey in 1992 indicated that most schools of public health were engaged with governmental public health agencies in the areas of research, practica for students, and technical assistance, although without formal written agreements (36). By 1997, the investigators reported an overall increase in engagement between academia and public health practice, particularly in formalizing relationships through the establishment of public health practice advisory committees (62). In their final report in 1999 (31), the UIC team noted the most significant trends over the course of multiple surveys, including a steady increase in faculty appointments for public health agency staff, appointments of practica coordinators at schools, student interactions with public health agencies, and joint research.

## EARLY QUALITATIVE AND DESCRIPTIVE FOCUS ON ACADEMIC–PRACTICE LINKAGES

The *Journal of Public Health Management and Practice* (*JPHMP*) has published—in 2000, 2006, and 2014—three themed issues focused on academic–practice linkages. Most of this work is descriptive or observational (as opposed to experimental) and focused mostly on single AHD partnerships. The 2000 themed issue is notable for publishing the article mentioned earlier by Keck, which included the first known use of the term academic health department. A centerpiece of the 2000 themed issue was the report of the Council of Public Health Practice Coordinators (the Practice Council) of the Association of Schools of Public Health (ASPH, as it was then known; now the Association of Schools and Programs of Public Health, or ASPPH), entitled “Demonstrating Excellence in Academic Public Health Practice,” which focused on “academic public health practice” (4). The report identified the roots of the separation between academia and practice in the establishment of the first schools of public health, which emphasized discipline-focused research over the needs of the public health workforce. The authors defined “academic public health practice” as the “applied, interdisciplinary pursuit of scholarship in the field of public health” (4, p. 10). In particular, the scholarship of application would be most relevant when academicians turned their attention to the actual practice of public health, e.g., through governmental public health agencies. Thus, if the Practice Council’s report provided a clear definition of what was needed to bridge the disconnect between academia and practice—applied, interdisciplinary pursuit of scholarship in the field of public health—Keck’s article provided a path for the how: Academic public health practice could be operationalized through the mutually beneficial partnership that Keck named the academic health department.

The second *JPHMP* themed issue on AHDs in 2006 highlighted the considerable progress made in establishing AHD partnerships since the 2000 issue. Most notably, Conte et al. (16) reported on a CDC-funded effort in 2003 to support the establishment of AHDs. Twenty-three teams of schools of public health and health departments applied for $100,000 in funding for one year, and 14 were subsequently funded. Conte et al. reviewed 22 of the 23 applications, summarizing their approaches to strengthening academic–practice engagement. Conte et al. identified several common themes across the 22 applications, including

- the critical importance of achieving a balance of power between institutions,
- the challenges posed by large cultural differences between academia and practice,
- the importance of being problem driven as opposed to theory driven,
- the importance of strong leadership,
- the value of cultivating boundary-spanning agents, and
- the value of incentives (e.g., the availability of seed money for collaborative research, tuition support).

While many of these approaches and themes can be identified in the subsequent establishment and operations of AHD partnerships, this CDC-funded project was self-limited, and no final report or evaluation could be located.

The ASPH Practice Council provided a second “demonstrating excellence” report as a follow-on to their 2000 report noted earlier, with a focus on “Practice-Based Teaching for Public Health” (5). The authors of the report were very clear: “Without incorporating practice into a public health curriculum, public health education lacks purpose” (5, p. 21). The report included eight principles of practice-based teaching and provided a succinct definition: “Practice-based teaching is the applied, interdisciplinary pursuit of scholarly teaching to inform and enhance professional public health education and training” (5, p. 17).

The 2006 special themed issue also includes DiMaggio and colleagues’ (20) article describing the New York City Department of Health and Mental Hygiene and the Columbia University Mailman School of Public Health’s National Center for Disaster Preparedness, described earlier. Of particular importance is the article by Maeshiro (51), which reported on the Association of American Medical Colleges’ (AAMC) cooperative agreement with the CDC to establish the Regional Medicine–Public Health Education Centers (RM-PHEC) in 2003. Seven schools of medicine were funded to establish or enhance partnerships with a state or local health department (LHD) to improve the integration of public health concepts into medical curricula that would benefit all medical students.

Giving further emphasis to these other types of academic–practice partnerships, the 2006 *JPHMP* issue included an article by Swain et al. (66), describing the partnerships between the City of Milwaukee Health Department and the Medical College of Wisconsin—where two physicians from the medical school provided medical supervision and brought public health leadership through research and teaching—and between the Monroe County (New York) Department of Public Health and the University of Rochester Schools of Medicine and Dentistry and Nursing, collectively named the Center for Rochester’s Health. In Rochester, New York, the Center for Rochester’s Health led numerous academic–practice partnership activities, including health professional education to create a more culturally competent workforce; the Racial and Ethnic Adult Disparities in Immunization Initiative to address health disparities in adult immunizations; and the Finger Lakes Office of Surveillance and Epidemiology, which provided consultation, assistance, and training to staff in nine public health departments.

The 2014 special themed issue of *JPHMP* provides numerous examples of both new and well-established AHD partnerships, in Albany, New York (9); Seattle and King County, Washington (37); Ohio and Northern Kentucky (41); Knoxville, Tennessee (35); Johnson City and Sullivan County, Tennessee (10); Duval County, Florida (48); North Central Georgia (67); and Columbia, South Carolina (64). Lee and colleagues (46) also provided an update on the AHD partnership in Ohio that was established by Keck, as described above.

These AHD partnerships include a mix of state and local public health departments; they are governed by both formal and informal agreements; the operational and coordination mechanisms range from having a full-time staff person equally shared between the two entities, to designating a doctoral student to coordinate the AHD as a practicum, to establishing steering committees comprising both academic and practice representatives, without additional personnel. The primary focus of these AHD partnerships was the education of public health students, including field-based internships and practica, and on workforce development, whereas practice-based research was less common. Perhaps the most remarkable of these AHD partnerships is the School of Public Health, University at Albany and the New York State Department of Health, where the school’s original faculty members were health department employees who continued to work for the New York State Department of Health while taking on new roles as faculty (9). Finally, Neri et al. (55) and Akintobi et al. (1) provide articles on the special academic–practice relationships between CDC-funded Prevention Research Centers (PRCs) and both public health and medical academic units.

In summary, from 2000 to 2006 and then to 2014, these special themed issues of *JPHMP* provided the foundational elements of AHD partnerships, then documented the first funded AHD partnerships through the CDC and the AAMC, and finally included case study–like representations of a burgeoning number of AHD partnerships being established in a wide variety of settings and contexts.

## RESEARCH FOCUSED ON GROUPS OF AHDs

Midway through the span of these *JPHMP* special themed issues, Livingood et al. (50) reported on partnerships between academia and public health practice across 67 county health departments (CHDs) in Florida (see Table 1). The investigators sought to understand both the extent of academic–practice engagement at the CHD level (as opposed to state public health agencies) and the value that such relationships provided from the CHD’s perspective. More than 80% of the responding CHD administrators reported the existence of formal agreements with academic institutions such as colleges or universities, including junior and community colleges. The study revealed that the origins of academic–practice relationships were in workforce development, i.e., responding to the inadequacies of formal public health training among CHD staff and providing students with field experience. While there were only 4 accredited academic public health programs and 1 accredited school in Florida at the time of this study, administrators reported agreements with more than 50 distinct academic institutions. The added value of academic–practice linkages from the CHD perspective included enhancing the capacity of the local public health system to serve its communities, advancing the mission of the CHDs, and providing the core functions of public health.

Livingood’s interest in AHD partnerships stemmed from his experiences at the Duval County Health Department in Florida, which had established an academic–agency partnership with the University of Florida/Jacksonville Medical Center in the mid-1990s and which eventually expanded to include the University of North Florida and Florida A&M University (49). A large number of university faculty (32 in 2007) were contracted to serve in full-time Health Department positions, providing a comprehensive array of health care services. A significant element of this partnership was the Institute for Health, Policy, and Evaluation Research, which included centers for health statistics, evaluation research, community health research, policy research, and health services research for vulnerable children. Livingood and colleagues conducted an economic impact analysis of this AHD partnership in 2007, estimating that an additional 275 jobs in the county could be attributed to the AHD partnership, with a direct economic impact of $22 million. The full economic impact—direct, indirect, and induced—was estimated to be $41.8 million. Despite the very powerful evidence of benefit and impact, a change in governors showed just how vulnerable public health agencies are to political change. A new governor asserted greater authority over CHDs (even changing the name from county to state health department in each county), resulting in the termination of most collaborations between the health department in Duval County and its academic partners, including the elimination of the agency’s research institute and severe cutbacks in primary care (W. Livingood, personal communication, January 2020).

Two studies in the mid-2010s mirrored the Handler surveys from the 1990s in seeking both public health agencies’ and academic institutions’ perspectives on the added value of AHD partnerships with respect to public health system quality and performance, accreditation, research, and health reform. In 2015, Erwin et al. (22) surveyed the AHD learning community, which then included 338 members, comprising a mixture of established AHD partnerships as well as academicians, practitioners, and others interested in learning more about AHD partnerships. A second study in 2016 focused on public health academic institutions, comprising 53 schools and 103 programs that were accredited by the Council on Education for Public Health (CEPH) (25). The results of these two complementary studies are summarized in Table 1.

During this same timeframe, Brownson and colleagues conducted a series of studies focusing on evidence-based public health (EBPH), including evidence-based decision-making (EBDM) in governmental public health agencies (12, 24). The work on EBPH and AHD partnerships intersected with a 2019 study that measured the extent to which the presence of an AHD partnership influenced the provision of EBPH in LHDs (28). This study was among the first empirical investigations to provide evidence that AHD partnerships mattered. From a cross-sectional survey of 579 LHDs, with 376 valid responses (response rate = 65%), Erwin et al.(28) identified 192 LHDs (51.6%) that had a formal AHD partnership (defined as having a formal written partnership agreement, shared staff, or shared financial resources). LHDs that were engaged in AHD partnerships were more likely to report receiving support for using EBDM and were twice as likely to implement evidence-based interventions compared with those that had no AHD partnerships. Erwin et al. also found an increasing strength of association between EBPH and presence of an AHD partnership across the AHD partnership continuum, from no AHD partnership, to informal AHD partnerships, to formal AHD partnerships. This association suggests that for LHDs with no AHD partnership, even establishing informal relationships with academicians may provide benefits and serve as a springboard for the development of formal partnerships. Because this study was cross-sectional, however, no temporal associations could be made between the establishment of AHD partnerships and the support for EBPH.

## RELATED ACADEMIC–PRACTICE COLLABORATIONS

We have highlighted examples of academic–practice partnerships, such as the PRCs, that, while not labeled as AHD partnerships, clearly have functioned in a similar capacity. In this section, we describe several of these partnerships, some of which have gone by the wayside while others are ongoing. While these are summarized in Table 2 and briefly described below, it is beyond the scope of this article to show the extensive amount of research coming from or about these examples.

The PRCs were authorized by Congress in 1984 and are funded and managed through the CDC (https://www.cdc.gov/prc/about-prc-program/index.htm). The authorization required PRCs to be established in either accredited schools of public health or schools of medicine with a preventive medicine residency program. The original purpose of PRCs was to understand how chronic diseases or conditions can be prevented or mitigated by conducting and applying community-engaged research, and thus a key feature of PRCs is the establishment and support of community partnerships. These partnerships often involve governmental public health agencies at the state or local level, giving them the appearance of an AHD partnership. A complete index of PRC research findings is available on the PRC website (https://www.cdc.gov/prc/study-findings/index.html).

Public Health Training Centers (PHTCs) were established by the HRSA in 1999 to provide training and expand the competencies of the public health workforce at the state, local, and tribal levels (8). Originally structured to focus on competencies for achieving the health objectives for the nation (e.g., Healthy People 2000), the PHTCs were reorganized in 2013 to include training for implementing the public health features of the Patient Protection and Affordable Care Act. There are now 10 regional PHTCs, one in each federally designated public health region; each center involves schools of public health across each region to work directly with local, state, and tribal public health departments.

Academic Centers for Public Health Preparedness were established by the CDC in 2000; these were subsequently replaced by the Preparedness and Emergency Response Learning Centers (PERLCs) as companion programs to the Preparedness and Emergency Response Research Centers (PERRCs) (40). The PERRCs were established to support applied public health systems research to strengthen and improve national public health preparedness and emergency response capabilities, whereas the PERLCs were aimed at enhancing the public health workforce competencies in emergency preparedness and response. A 2014 special supplement issue of *Public Health Reports* provided further documentation of the activities and value of the PERRCs (61).

In 2003, RM-PHECs were funded by the CDC through the AAMC to improve the integration of public health, population health, and prevention education into medical school curricula and to develop new field opportunities for medical students at public health agencies (51). In 2010, the AAMC and the CDC convened a conference on “Patients and Populations: Public Health in Medical Education” to help disseminate lessons learned by the RM-PHECs, and a 2011 special supplement of the *American Journal of Preventive Medicine* featured several articles based on the conference presentations (52).

From 2007 to 2015, the Robert Wood Johnson Foundation (RWJF) funded the Public Health Practice-Based Research Network (PBRN) to support teams of academicians and practitioners to conduct research on the organization, financing, and delivery of public health services and to apply research findings to improve both public health practice and policy. The program funded 12 networks and supported the work of 16 affiliate networks through technical assistance and access to other RWJF funding opportunities. As of 2013, 926 LHDs, 20 state agencies, and 35 academic units had been involved in PBRN-funded studies (54).

## AHDs IN THE CONTEXT OF COVID-19

Washington state—ground zero for the first confirmed COVID-19 case in the United States—provides an example of an academic–public health practice partnership that used innovation to facilitate daily situation reports to guide EBDM. At the onset of the pandemic, Washington Department of Health (WA DOH) epidemiologists created a daily literature situation report (Lit Rep) within the incident management team to inform decision makers of the best available evidence during the rapidly evolving response. However, by May 2020, the number of relevant daily publications outpaced the epidemiologists’ capacity, so the WA DOH contracted with the University of Washington Alliance for Pandemic Preparedness to assemble a team of 2 faculty and 12 students to create the daily Lit Rep as well as special in-depth reports requested by public health leaders (63).

In April 2020, the National Association of County and City Health Officials (NACCHO) called for a “massive expansion of professionals and trained volunteers equipped with the appropriate skills, training, and technology, distributed equitably across the country to help identify, notify, and support those who may have been exposed [to COVID-19], and help them self-quarantine to stop the spread” (53, p. 1). In response, existing academic–practice partnerships, including public health, medical, and secondary education, generated surge public health workforce capacity for contact tracing and case investigation in collaboration with local and state departments of health and academic medical systems (6, 19, 42, 45, 47, 56, 57). We summarize key successes and lessons learned.

Yale University coordinated ~50 student (public health, medicine, and nursing) volunteer contact tracers in close partnership with local and state (Connecticut) departments of health to contact trace for the university and for New Haven, Connecticut. The health department created an interview tool using Veoci, a local emergency management software platform, and Yale created a secure database to store information. During summer 2020, the Maricopa County Department of Public Health asked Arizona State University’s Student Outbreak Response Team to provide surge capacity for contact tracing and case investigation (45). Similarly, in Ohio, the presence of a formal academic–public health practice partnership created the necessary preconditions that enabled rapid, mutually beneficial collaboration, whereby academic and health department partners combined resources, resulting in improved public health (47). Ohio University’s strengths included workforce capacity (i.e., student volunteers) and advanced information technology infrastructure, while the health department brought infectious disease expertise and knowledge of changing guidelines. In New York, undergraduate nursing students partnered with the Long Island Health Department to conduct contact tracing as their senior capstone experience (19). The new capstone provided surge capacity for the health department, and nursing students reported that they gained a sense of purpose and value for communication, education, and teamwork. While these academic–practice partnerships helped meet NACCHO’s call for a strong, fast influx of public health workers and volunteer contact tracers, it is not clear whether the surge workforce was distributed equitably across the United States.

## AHDs, STRUCTURAL RACISM, AND HEALTH EQUITY

Given the AHD focus on workforce development, student experiences, and practice-based research, we believe that AHDs are uniquely positioned to advance public health’s efforts to address structural racism and promote health equity, both in academic public health and in public health practice. Health equity means that everyone has opportunity to reach their fullest potential for health. As Kumanyika (43) and Brownson et al. (11) describe, a health equity framing moves us away from the deficit mindset of disparities—what society is doing poorly—to a positive focus on what society can achieve. While we focus primarily on race and racism, the principles apply to a wide range of issues of importance and relevance to public health practice (e.g., LGBTQ equity, disability rights).

Structural racism, a barrier to health equity, refers to a “system in which public policies, institutional practices, cultural representations, and other norms work in various, often reinforcing ways to perpetuate racial group inequity” (44, p. 11). Despite the APHA recognizing racism as a fundamental cause of racial health disparities in 2001 (3), Chandler and colleagues’ (15) systematic review found only 11 peer-reviewed articles published between 2006 and April 2020 that described teaching methods and curriculum that frame racism as a structural determinant of health, and none of the 11 examples involved collaboration with a local or state health department partner.

The New York City Department of Health’s (NYCDH) Bureau of Communicable Diseases implemented a Dismantling Racism (DR) intervention, a seven-pronged approach rooted in Ford & Airhihenbuwa’s (30) Public Health Critical Race Praxis to address the internal, interpersonal, and institutional levels of racism in the health department. Duerme and colleagues (21) describe the rationale, implementation, and evaluation of the DR intervention, as well as recommended tactics for other public health agencies. While the NYCDH’s intervention was health department led, Duerme and colleagues (21) reference academic public health in two ways: first, the microaggressions report (the basis for interventions) acknowledged that white student interns, especially those connected to elite schools and social networks, had been privileged unfairly; and second, interns were invited to participate in the health department–led interventions.

**Figure 1:**
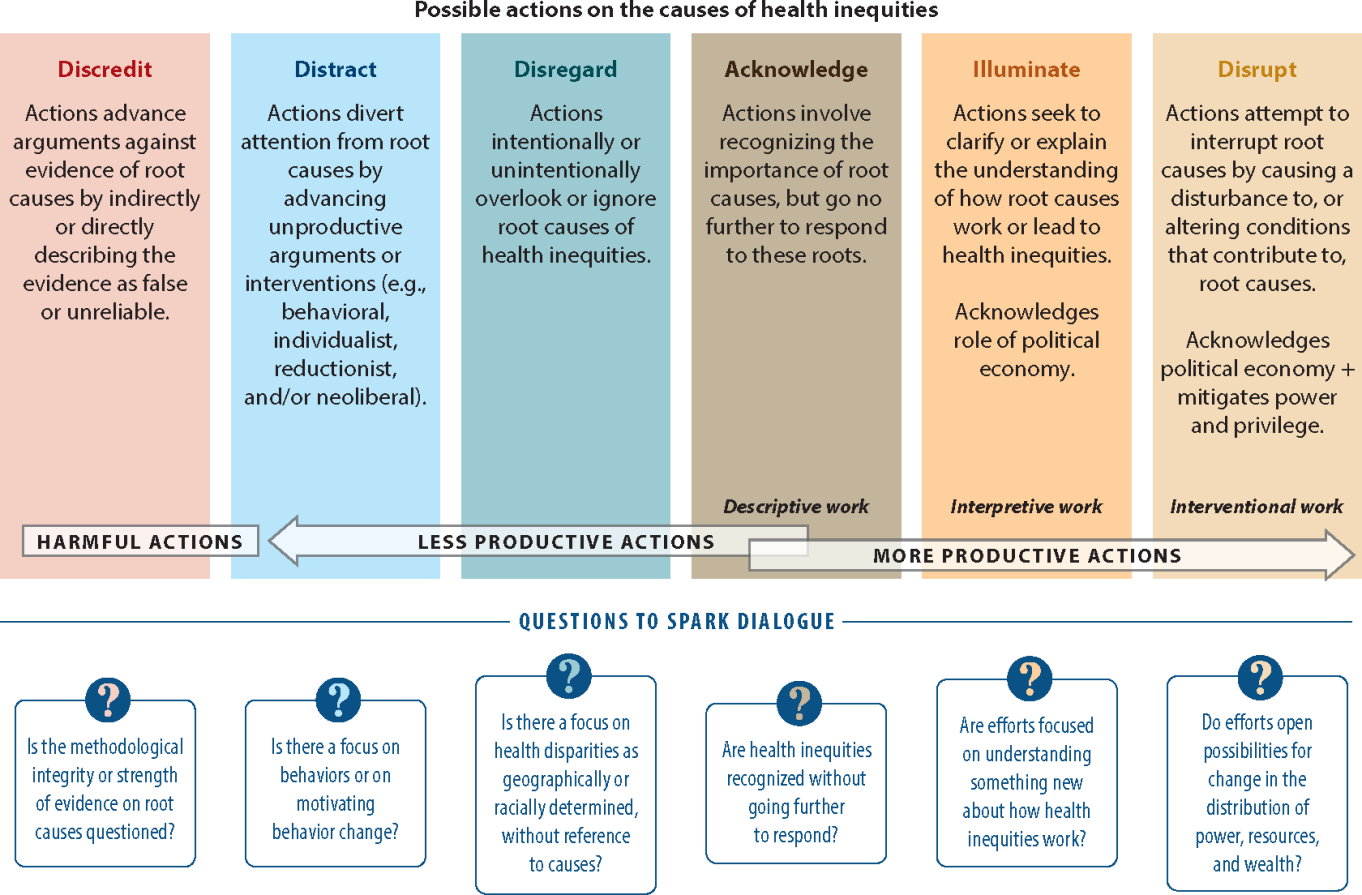
Possible actions in response to evidence about causes of health inequities. Figure adapted from Reference 58 (CC BY 4.0).

Alang and colleagues (2) offer strategies to address structural racism and white supremacy through public health’s three core functions: assessment (e.g., track police brutality and exposure to structural racism), policy development (e.g., educate policy makers on white supremacy indicators and the influence of white supremacy on social determinants of health), and assurance (e.g., assure racial equity competency among students and practitioners and use critical race theory to inform teaching, research, and practice). Recognizing the discordance between good intentions, good evidence, and meaningful action vis-à-vis health equity, Plamondon and colleagues (58, 59) provide a theoretically informed but very simple tool for assessing the alignment between knowledge and action for health equity. The tool presents six possible actions that describe ways in which efforts to advance health equity could address causes of health inequities: discredit, distract, disregard, acknowledge, illuminate, or disrupt (see Figure 1).

AHD partnerships are well-positioned to address structural racism as a fundamental cause of health inequities, and they can also advance health equity for other marginalized and vulnerable populations, including LGBTQ+ communities, impoverished communities of all races and ethnicities, and migrant and immigrant communities (see the sidebar titled Fostering a Diverse Workforce on the role of the AHD in promoting workforce diversity). Brownson et al. (11) provide 10 recommendations for advancing the positive framing of health equity; although the focus is on implementation science, the relevance for AHD partnerships is quite clear, given their applied practice orientation: (*a*) link social determinants with health outcomes, (*b*) build equity into all policies, (*c*) use equity-relevant metrics, (*d*) study what is already happening, (*e*) integrate equity into implementation models, (*f*) design and tailor implementation strategies, (*g*) connect to systems and sectors outside of health, (*h*) engage organizations in internal and external equity efforts, (*i*) build capacity for equity in implementation science, and (*j*) focus on equity in dissemination efforts.

## RESEARCH TENSION AND POTENTIALITIES

Research within an AHD context may present several areas of tension, while also offering potential “solutions”; here we focus on three of these areas.

First, we consider the general problem of the settings in which evidence is generated, and how, and thus the value and utility of that evidence. Given the relative paucity of the evidence of impact for what public health departments do, we repeat what has become a mantra for practice: If we want more evidence-based practice, we need more practice-based evidence (32). The AHD partnership is the ideal context for striking a balance between explicit (research) knowledge and tacit (lived experience) knowledge (13).

Second, there is often a significant gap between academicians’ research emphasis and the research questions that are of greatest interest to the practitioner. The academician is often more motivated by what can be supported by extramural funding, which, from a practitioner’s perspective, is often narrow and of less relevance to what is actually encountered in everyday practice. Of more interest to the practitioner is simply whether a particular program is achieving its intended impact, which can become evaluation research. A balance can be sought: providing the academician access to public health data to pursue their research interests, while “paying” for such access by assisting practitioners with answering their questions of interest and concern. In addition, the time frame for research also contributes to the research–practice gap—research grants often take years to obtain and carry out, whereas practice and policy move much more quickly. The AHD is a rich arrangement for addressing many of the challenges in the research translation pipeline.

Third, and closely related to the second, is the tension between internal and external validity in research design and implementation. The academician is often most concerned about internal validity: Is the research properly designed and conducted to be able to answer specific questions? As described by Green et al. (33), “[T]he more highly controlled the studies producing evidence with strong internal validity (controls on confounding variables and selection bias) are, the more they might have squeezed out some of the external validity of that evidence” (p. vi). Working in the context of an AHD partnership offers an opportunity to consider more carefully how academic research can attend to issues of external validity and relevance at earlier phases of the research translation pipeline and accelerate the translation of research into practice. AHD partnerships can also increase the internal validity of practice-based research with elements such as the use of reliable/valid measures, grounding the work in theory. Again, there is a balance to be sought, and the AHD partnership context can offer such opportunities; in practice-based research, the academician can retain their focus on internal validity while allowing the real-world experiences of practitioners to influence the level of external validity. Given that AHDs operate across a variety of ever-changing local and state policies, natural experiments involving AHDs offer a robust, timely methodology with which to study differences in population health outcomes and programs across states (e.g., abortion laws, Medicaid expansion, firearm regulations), counties, and cites (e.g., police accountability, mental health resources), as well as the differential impacts of upstream forces such as affordable housing and bans on teaching critical race theory.

## A SUMMARY OF AHD PARTNERSHIP-RELATED RESEARCH

Our own exploration of AHD partnerships has focused on the very basic question, what is the evidence indicating that these academic–practice partnerships matter? To guide this work, we created a logic model for evaluating AHD partnerships (27; see Supplemental Figure 1), which then served as the basis for developing a research agenda for AHD partnerships (23) (see Supplemental Table 1). This research agenda is available on the Public Health Foundation’s website (http://www.phf.org/resourcestools/Pages/AHD_Research_Agenda.aspx). Using the logic model, we can summarize the research on AHD partnerships vis-à-vis inputs, outputs, outcomes, and impact as follows.

In terms of inputs,

- Formal relationships based on written agreements are important, but informal agreements are still better than no relationship;
- shared personnel help to bridge the distinct cultures of academia and practice and can build strong ties; and
- academic and practice partners can jointly gather and track data for enhancing education, research, and service.

Outputs include the following:

- the development of evidence-based practices in governmental public health agencies is more strongly associated with settings that have AHD partnerships than with settings that do not have AHD partnerships;
- AHD partnerships can enhance the translation of research into practice and inform the field of dissemination and implementation science;
- practice-based research can be published in peer-reviewed journals and in textbooks; and
- AHD partnerships can inform the development, delivery, and evaluation of academic curriculum and public health services.

Outcomes in this logic model can be summarized as follows:

- health departments participating in AHD partnerships implement evidence-based practices to a greater degree than do health departments that do not participate in AHD partnerships;
- AHD partnerships that involve medical students and residents can serve as models for integrating public health and prevention education into medical school curricula;
- AHD partnerships can yield a significant return on investment;
- AHD partnerships can enhance workforce development; and
- AHD partnerships can improve the competencies of students, practitioners, and academicians.

Finally, the impact of AHD partnerships is that

- AHD partnerships can facilitate the mission of academic institutions and of public health agencies.

## CURRENT AND FUTURE RESEARCH AND PRACTICE

Our current research on AHD partnerships is to understand how to leverage structures and processes of AHD partnerships to facilitate implementation of cancer-related evidence-based programs and policies. We are conducting a study to understand more about how successful partnerships are structured and to experimentally evaluate the effect of strategies to strengthen AHD partnerships and, in turn, improve the implementation of evidence-based cancer control and prevention programs and policies. A mixed-methods approach will be used to evaluate changes in AHD partnerships and to understand how contextual factors may have impacted the AHD partnership’s ability to support the adoption of evidence-based programs and policies. That such work is supported by National Institutes of Health (NIH) funding (R01CA262011, Mazzucca-Ragan, principal investigator) is an indication of how far the field of research on AHD partnerships has advanced.

We encourage AHDs to study the NYCDH’s multilevel approach to applying Public Health Critical Race Praxis to plan, implement, and evaluate dismantling racism within health departments and academic public health. Even in settings that are markedly different from New York, reflecting on the Public Health Critical Race Praxis can start a conversation among members of AHD partners about how DR can be operationalized in their communities. AHD partnerships are also well-positioned to implement Dent and colleagues’ (18) recommendations to educate students and practitioners on historical, embedded racism in the United States; to create policies against racism to improve organizational climate; to examine hiring and promotion to increase diversity among leadership positions; to invest in historically black colleges, Hispanic-serving institutions, and tribal-associated schools; and to study pipeline programs for historically excluded groups. AHD partnerships are well-positioned to apply Plamondon’s tool (Figure 1) to assess alignment between knowledge and action for health equity, and we strongly recommend this approach as a means for productive action in addressing racism as a public health crisis.

## CONCLUSION

AHDs, and related academic–practice partnerships, provide important opportunities to strengthen the evidence base for public health practice and improve the relevance of public health curriculum and academic offerings in addressing the real-world challenges faced by governmental public health agencies. Students are at the core of the AHD partnership, as they are the ones to complete academic degrees within the setting of an AHD, and they can become the public health workforce of tomorrow, better equipped to identify and apply the evidence base for practice. COVID-19 has provided additional opportunities for academic–practice engagement, including practice-based research that can explore, for example, the barriers to enhancing vaccine uptake, the relevance of which will extend far beyond COVID-19. The pandemic has also further revealed the ongoing challenges of addressing structural racism and assuring health equity. We believe AHD partnerships can play increasingly important roles in overcoming these challenges through enhanced capacities to positively influence the social determinants of health at all levels of governmental public health practice. Research is underway to determine the ideal set of skills and supports that AHD partnerships need for making sustainable progress.

## Supplementary Material

Supplemental Table and figure

## Figures and Tables

**Table 1:** Summary of empirical studies on AHD partnerships

Study	Focus of study	Respondents	Key findings
Livingood et al. 2007 (49)	County health departments in Florida (*n* = 67)	51 county health department directors (response rate = 76%)	◾ Most reported formal agreements, involving 50 different academic institutions. ◾ Most focused on workforce development.
Erwin et al. 2016 (22)	AHD learning community (*N* = ~265 members as appropriate targets)	110 responses (response rate = 42%); mixture of academicians, practitioners, others	◾ 65 (59%) report that their organization— either an academic institution or a governmental health agency—participated in an AHD partnership. ◾ Most frequently cited characteristics of the AHD partnership included collaborative public health education/training (85%) and joint research projects (64%). ◾ Approximately half of the respondents described a formal written partnership agreement such as a memorandum of understanding, letter, or agreement or a contract.
Erwin et al. 2016 (25)	53 CEPH-accredited schools and 103 CEPH-accredited programs, for a total of 156 institutions	117 responses (response rate = 75%)	◾ Approximately half of the academic institutions indicated that they had an AHD partnership with a governmental health agency, with the majority of these having a formal written agreement. ◾ More than 70% of the respondents indicated that their public health practice partner actively recruited their graduates for employment. ◾ Academicians reported substantial involvement in the prerequisites for accreditation through the Public Health Accreditation Board.
Erwin et al. 2019 (28)	579 LHDs	376 valid responses (response rate = 65%)	◾ LHDs that were engaged in AHD partnerships reported support for using EBDM and were two times more likely to implement evidence-based interventions. ◾ There is an increasing strength of association between EBDM and AHD partnerships, from no partnership, to informal partnerships, to formal partnerships.

Abbreviations: AHD, academic health departments; CEPH, Council on Education for Public Health; EBDM, evidence-based decision-making; LHD, local health departments.

**Table 2:** Selected academic-practice partnerships in addition to AHD partnerships

Title	Primary partners	Supporting organization	Period of funding
PRCs	Accredited schools of public health or medicine with a preventive medicine residency	CDC	1984-present, with 26 PRCs currently funded
PHTCs	Accredited schools of public health and local and state health departments	HRSA	1999-present
PERLC; PERRC	Accredited schools of public health	CDC, managed by the ASPH (now known as the ASPPH)	2000–2015
RM-PHECs	Medical schools, required to partner with at least one state or local health department	CDC, through the AAMC	2003–2012
PBRNs	Accredited schools or programs of public health, in partnership with state or local health departments	RWJF	2007–2015

Abbreviations: AAMC, Association of American Medical Colleges; ASPH, Association of Schools of Public Health; ASPPH, Association of Schools and Programs of Public Health; CDC, Centers for Disease Control and Prevention; HRSA, Health Resources and Services Administration; PBRN, public health practice-based research network; PERLC, preparedness and emergency response learning centers; PERRC, preparedness emergency response research centers; PHTC, public health training center; PRC, Prevention Research Center; RM-PHECs, regional medicine-public health education centers; RWJF, Robert Wood Johnson Foundation.

## AHD EXAMPLES FROM OTHER COUNTRIES

While the term academic health department tends to be used mainly in the United States, other countries have analogous processes and partnerships. For example, in Australia, some strong academic–government partnerships have embedded researchers within public health agencies, in part to foster evidence-based decision-making (68, 69). In addition, cofunded government and research entities facilitate public health policy–oriented research (e.g., the Australian Prevention Partnership Centre; https://preventioncentre.org.au/). In Canada, there is a decades-long history of partnership and collaboration between local health units and academe. For example, teaching health units were established to provide formal affiliations between public health practice and academic health sciences centers (14).

## FOSTERING A DIVERSE WORKFORCE

The country’s workforce turnover is an opportunity for public health to hire and promote underrepresented minority individuals and other underrepresented groups (e.g., LGBTQ+, disabled people) among all levels, particularly leadership positions. During the initial year of the COVID-19 pandemic, at least 181 state and local health department leaders left their positions (7). Among a nationally representative sample of 45,000 state and local health department staff surveyed between September 2021 and January 2022, more than 1 in 4 were considering leaving (17). In public health settings, just as it is in the private sector, workplace culture is a strong predictor of employee turnover [see, e.g., Sull and colleagues’ analysis of factors driving the “Great Resignation”—the sharp increase in employee resignations in response to the COVID-19 pandemic (65)]. Beyond what should be of importance to all LHDs, what is the added value of an AHD partnership in stimulating a healthy workplace culture that welcomes, values, and affirms underrepresented groups and buffers stress and burnout? In what ways can AHD partnerships train, hire, promote, and retain a more diverse workforce and develop healthy, equity-minded workplaces?

## SUMMARY POINTS

1. AHDs are mutually beneficial partnerships between public health academia and local or state health departments.
2. AHDs focus primarily on workforce development, education and practice-focused teaching, and practice-based research.
3. AHDs may have joint (shared) personnel.
4. Health departments with formal AHD partnerships are twice as likely to implement evidence-based interventions compared to health departments without AHD partnerships.
5. AHDs provided surge capacity for contact tracing and case investigation during theCOVID-19 pandemic.
6. AHDs have the potential to illuminate and disrupt structural racism, particularly through implementation science with a health equity lens, public health critical race praxis, and Plamondon and colleagues’ tools to assess alignment and action for health equity.

## FUTURE ISSUES

1. What opportunities and challenges does the workforce turnover present for public health academia and practice, and how will AHDs work together toward rejuvenating and refocusing the workforce, both within public health and across other sectors?
2. How will AHDs create conditions for racial justice and health equity, particularly within the public health workforce and among partners?
3. Which skills and supports do AHD partnerships need for making sustainable progress in positively influencing the social determinants of health?
4. Given the deeply polarizing politicization of public health amid the COVID-19 pandemic, in what ways will academic–practice partnerships contribute to rebuilding the public’s trust in public health?
5. How might tools from implementation science offer AHDs an applied methodology to tailor evidence-based interventions to better fit community context?
6. How might the body of research on AHDs move toward stronger study designs?
