# Supplementary material for: The Value and Impacts of Academic Public Health Departments
Source: Annu Rev Public Health. Author manuscript; Available in PMC 2025 Mar 20. (PMC11925481; doi:10.1146/annurev-publhealth-071421-031614)
Supplement: Supplemental Table and figure [file NIHMS2058555-supplement-Supplemental_Table_and_figure.pdf]

Supplemental Material: Annu. Rev. Public Health 2023. 44.
https://doi.org/10.1146/annurev-publhealth-071421-031614
The Value and Impacts of Academic Public Health Departments
Erwin, Grubaugh, Mazzucca, and Brownson

Supplemental Figure 1. A logic model for evaluating academic health departments. Figure reproduced from Erwin et al. (29) with permission from Wolters Kluwer Health, Inc. (CC BY-NC-SA 2.0).

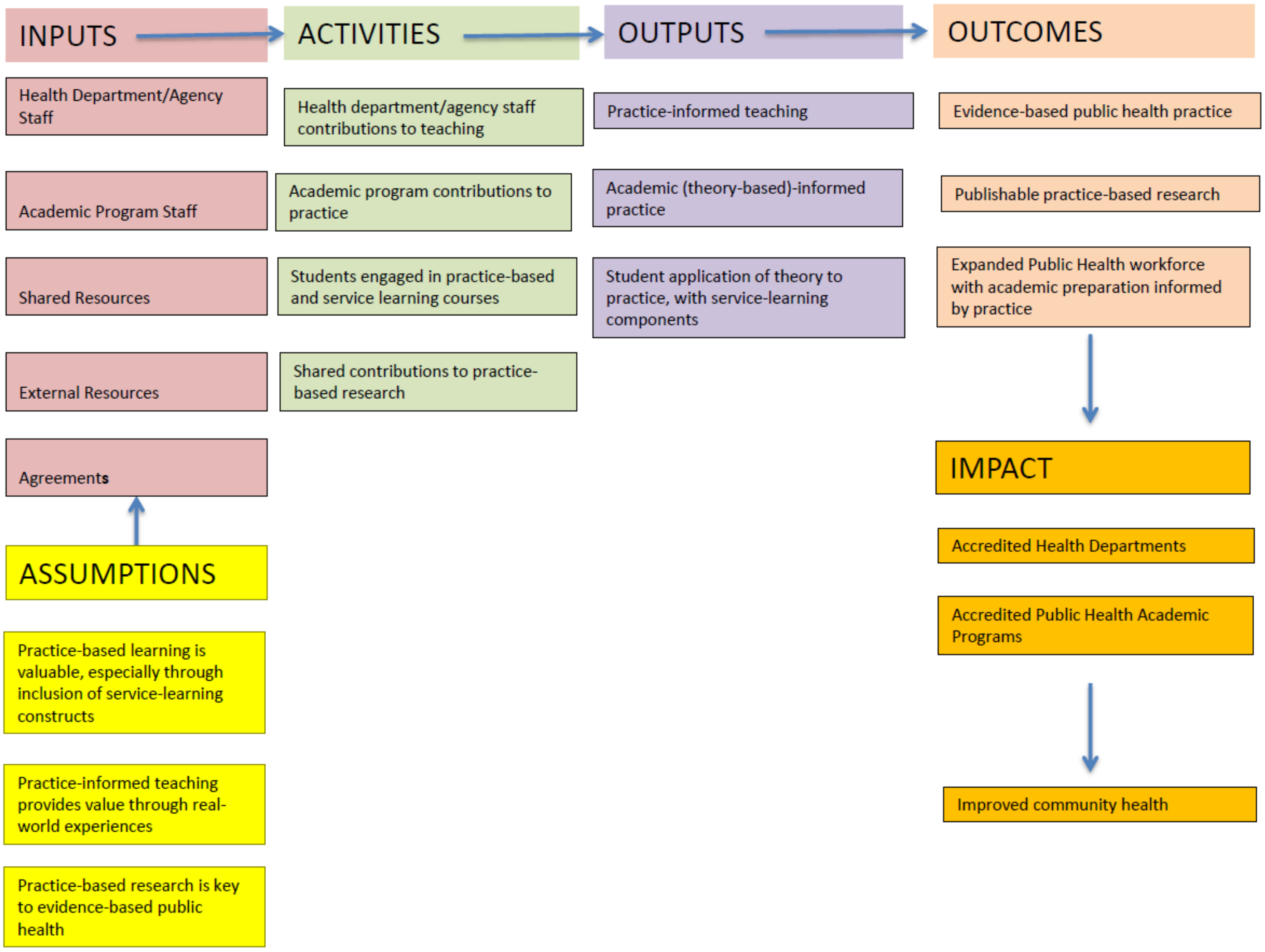

Downloaded from www.annualreviews.org. Guest (guest) IP: 140.177.67.63 On: Sat, 08 Mar 2025 03:36:25

Downloaded from www.annualreviews.org. Guest (guest) IP: 140.177.67.63 On: Sat, 08 Mar 2025 03:36:25

Supplemental Table 1. Academic Health Department Research Agenda. From Erwin PC, Brownson RC, Livingood WC, Keck CW, Amos K. 2017. Development of a Research Agenda Focused on Academic Health Departments. *Am. J. Public Health* 107:1369–75, used with permission of the *American Journal of Public Health*

Logic Model Parameter	Potential Research Questions
**Inputs**	1. What models and theories of education and training are most effective in creating the conditions to establish AHDs?
	2a. What styles of leadership are most effective in establishing and sustaining AHDs?<br>2b. What styles of management are most effective in establishing and sustaining AHDs?
	3a. How do practitioners in settings with AHD partnerships differ from practitioners in settings without AHD partnerships in terms of background, training, and expertise?<br>3b. How do academicians in settings with AHD partnerships differ from academicians in settings without AHD partnerships in terms of background, training, and expertise?
	4a. What are the critical resources for establishing AHDs?<br>4b. What are the critical organizational environments for establishing AHDs?
	5. What is the variability across AHDs in resources, and how does such variability matter?
	6. What is the value of shared personnel in AHDs?
	7. Which types of personnel contribute most to AHDs?
	8. What arrangements for sharing personnel in AHDs have been successful?
	9. What types of formal agreements have been used to establish AHDs?
	10. What are the critical elements of formal agreements that have been used to establish AHDs?
	11. How do the prevailing attitudes about practice and academia differ in settings with AHD partnerships vs. settings without AHD partnerships? Do these attitudes influence the ability to establish and maintain AHDs?
	12. What are the advantages to learning in AHD settings from the perspectives of students, faculty, and practitioners?

Downloaded from www.annualreviews.org. Guest (guest) IP: 140.177.67.63 On: Sat, 08 Mar 2025 03:36:25

	13. What are the advantages to working in AHD settings from the perspectives of faculty and practitioners?
	14. Are academic and practice organizations prepared to jointly develop data for enhancing teaching, research, and practice?
**Activities**	1. What value do AHDs add to service-learning courses?
	2. What are the mechanisms by which academicians contribute to program development, implementation, and evaluation in the practice setting?
	3. What are the mechanisms by which practitioners contribute to development, implementation, and evaluation of education and research in the academic setting?
	4. What are the ways in which AHDs facilitate practice-based research?
	5. Do AHDs enhance the quality and relevance of student field placements (internships), and if so, how?
	6. What roles do AHDs have in Accountable Care Organizations?
	7. What roles do AHDs have in addressing the social determinants of health, and in particular, health inequities?
	8. What roles do AHDs have in successful implementation of state and federal community benefit requirements?
**Outputs**	1. How does student competency development (related to the Core Competencies for Public Health Professionals) differ between students in learning environments with AHD partnerships and those in learning environments other than AHDs?
	2. How does the application of knowledge, both during their academic program and after graduation, differ between students in AHD settings and those in settings without AHD partnerships, from the perspectives of students, faculty, and practitioners?
	3. Does the presence of academicians impact the development of evidence-based practices in ways that are more effective and efficient in settings with AHD partnerships than settings without AHD partnerships?
	4. How does learning in AHD settings impact students' civic engagement?

	5. Do AHDs enhance the translation of research into practice, and if so, how?
	6. How can AHDs inform the field of dissemination and implementation science?
	7. Do AHDs lead to more and better partnerships (beyond the AHD partnership itself)?
	8. Does being engaged in AHD activities enhance the “standing” of academicians and practitioners in their fields?
	9. Does having faculty engaged through AHD partnerships enhance delivery of essential public health services, and if so, how?
	10. Does having faculty engaged through AHD partnerships enhance workforce development and training for the current public health workforce, and if so, how?
	11. Does having practitioners engaged through AHD partnerships enhance public health education, and if so, how?
	12. Does having practitioners engaged through AHD partnerships enhance public health research, and if so, how?
	13. What is the impact of AHDs on the development and delivery of academic curriculum?
	14. What is the impact of AHDs on the development and delivery of public health services?
	15. What is the impact of AHDs on the development and delivery of graduates well-prepared for careers in public health practice?
	16. Does involvement of practitioners in the classroom impact their practice?
	17. Does involvement of faculty in practice settings impact their teaching?
	18. Do AHD partners publish their work in peer-reviewed journals, in textbooks, or in other ways?
	19. Do AHD partnerships enhance the effectiveness of public health practice in advocating or defending policies before local or state legislative or oversight bodies?

Downloaded from www.annualreviews.org. Guest (guest) IP: 140.177.67.63 On: Sat, 08 Mar 2025 03:36:25

Downloaded from www.annualreviews.org. Guest (guest) IP: 140.177.67.63 On: Sat, 08 Mar 2025 03:36:25

**Outcomes**	1. Do health departments participating in AHD partnerships implement evidence-based practices to a greater degree than health departments that do not participate in AHD partnerships?
	2. Are students in AHD settings more successful in obtaining employment than students in settings without AHD partnerships?
	3. Are students with experience in AHD settings more likely to take jobs in health departments following graduation than students without such experience? To pursue careers in public health practice?
	4. Are health departments that hire students with experience in AHD settings more satisfied with their new employees compared to new hires without this experience?
	5. Are health departments that participate in AHD partnerships more successful in achieving accreditation through the Public Health Accreditation Board (PHAB) than health departments that do not participate in AHD partnerships?
	6. Are academic programs that participate in AHD partnerships more successful in achieving accreditation through the Council on Education for Public Health (CEPH) than academic programs that do not participate in AHD partnerships?
	7. Will AHDs that involve medical students and residents serve as models for patient-centered primary care?
	8. What is the return on investment for AHDs, from both the academic and practice perspectives?
	9. What is the impact of AHD partnerships on the skills and decision-making processes of health department leaders?
	10. What is the impact of AHD partnerships on the skills and decision-making processes of academic institution leaders?
	11. What is the impact of AHD partnerships on the organizational climate and culture of health departments?
	12. What is the impact of AHD partnerships on the organizational climate and culture of academic institutions?
	13. Do health departments with AHD partnerships operate more efficiently and effectively than health departments without AHD partnerships?

Downloaded from www.annualreviews.org. Guest (guest) IP: 140.177.67.63 On: Sat, 08 Mar 2025 03:36:25

	14. Do health departments with AHD partnerships demonstrate more effective financial allocation strategies than health departments without AHD partnerships?
	15. Do health departments with AHD partnerships perform better than those without AHD partnerships in assuring delivery of essential public health services to their communities?
	16. Do academic institutions with AHD partnerships perform better than those without AHD partnerships in assuring delivery of graduates well-prepared to enter the public health workforce?
	17. What are the critical elements for sustaining AHD partnerships?
	18. Do AHD partnerships enhance workforce development and training for public health practice?
**Impact**	1. Do AHD partnerships facilitate the achievement of the mission of the public health practice organization – assuring conditions in which people can be healthy?
	2. Do AHD partnerships facilitate the mission of the academic institution?
	3. Does the presence of AHD partnerships have a greater impact on community health improvement activities and outcomes than not having AHD partnerships?
